# Global, regional, and national burden and trends of rheumatoid arthritis among the elderly population: an analysis based on the 2021 Global Burden of Disease study

**DOI:** 10.3389/fimmu.2025.1547763

**Published:** 2025-04-15

**Authors:** Lu Wei, Xiang Chen, Mengmeng Liu

**Affiliations:** ^1^ Department of Trauma Center, Liuzhou Worker’s Hospital, The Fourth Affiliated Hospital of Guangxi Medical University, Liuzhou, China; ^2^ Guangxi Key Laboratory for the Research and Clinical Translation of Orthopedic Biomaterials, Liuzhou, China; ^3^ Department of Bone and Joint Surgery, the First Affiliated Hospital of Guangxi Medical University, Nanning, China; ^4^ School of Physical Education And Health, Guangxi Medical University, Nanning, China

**Keywords:** rheumatoid arthritis, elderly, disease burden, prevalence, incidence, DALYs

## Abstract

**Background:**

Rheumatoid arthritis (RA) is an autoimmune and inflammatory disease. In elderly patients, the disease progresses more rapidly, involves more complications, and places a greater burden on health. Currently, there is a lack of studies investigating the disease burden of RA in the elderly population.

**Methods:**

We analyzed data on elderly rheumatoid arthritis from the Global Burden of Disease (GBD) database for 1990–2021, focusing on three main indicators: prevalence, incidence, and Disability-Adjusted Life Years (DALYs). Percentage change and the estimated annual percentage change (EAPC) were used to evaluate the trends in the disease burden.

**Results:**

In 2021, the global prevalence cases, incidence cases, and DALYs of elderly RA were 7,919,136, 334,291, and 1,549,877, representing increases of 157.59%, 169.71%, and 116.53% compared to 1990. Both the prevalence rate and incidence rate increased, with EAPCs of 0.54 (95% CI: 0.5, 0.58) and 0.75 (95% CI: 0.7, 0.79), respectively. Notably, the prevalence rate in females was 2.2 times higher than that in males. The DALY rate showed a slight decline. Among the five Socio-demographic Index (SDI) regions, the High SDI region had the highest prevalence cases, incidence cases, and DALYs in 2021, with 2,821,305, 114,994, and 483,579, respectively, accounting for 36%, 34%, and 32% of the global totals. This region also recorded the highest prevalence and incidence rates. In contrast, the Low SDI and Low-middle SDI regions exhibited the fastest growth in both prevalence and incidence cases as well as rates. The highest prevalence cases and incidence rate were observed in the 65–69 age group. Decomposition analysis revealed that the rising disease burden was primarily attributable to the growth of the global elderly population.

**Conclusions:**

Between 1990 and 2021, the global burden of rheumatoid arthritis in the elderly population increased. The High SDI region experienced the highest disease burden. In contrast, the Low and Low-middle SDI regions showed the most rapid growth in disease burden. Females exhibited a higher burden compared to males, with the highest burden observed in the 65–69 age group. Early diagnosis and treatment in elderly patients are essential to mitigating adverse outcomes and reducing the burden.

## Introduction

Rheumatoid arthritis (RA) is a chronic autoimmune and inflammatory disorder with a globally varying prevalence ranging from 0.25% to 1%, predominantly affecting women. Key risk factors include smoking and genetic predisposition (1, 2). The primary clinical manifestations are joint pain, swelling, and deformity (3). If left untreated, the disease can progress to focal necrosis, adhesions, and fibrous proliferation on joint surfaces, which may ultimately result in joint ankylosis, severe deformity, and disability (4). Beyond the joints, rheumatoid arthritis can also affect extra-articular tissues and organs, including the heart, kidneys, lungs, digestive system, eyes, skin, and nervous system (5, 6). Notably, since 1990, the incidence and prevalence of rheumatoid arthritis have steadily increased, exacerbating its global disease burden and highlighting the need for enhanced prevention and management strategies (7, 8).

Notably, one-third of RA patients experience disease onset after the age of 60. Compared to younger patients, late-onset RA tends to progress more rapidly, affects a greater number of joints, leads to more severe systemic inflammation and complications, and results in a higher disability rate, thereby posing a significant health burden (9–11). Concurrently, the increasing aging population has contributed to a growing number of elderly RA patients, further exacerbating the disease burden (12). Although previous studies have summarized the disease burden caused by RA (13), there is currently no dedicated research focusing specifically on RA burden in individuals over 60. Therefore, investigating the burden and future trends of RA in elderly patients is of great importance, as it provides critical evidence to guide the development of public health policies.

The GBD study is a comprehensive research initiative designed to evaluate the epidemiological trends of various diseases and health conditions at global, regional, and national levels. By collecting and analyzing vast amounts of data, the GBD Study assesses the impact of diseases and associated risk factors on populations, providing essential guidance for public health policymaking, disease prevention, and health promotion (14, 15). In our study, we analyzed the incidence, prevalence, and DALYs of RA in elderly populations at global, regional, and national levels from 1990 to 2021. We further compared the distribution and trends of RA burden across different age groups and projected the incidence and prevalence of elderly RA for the next 20 years. These findings serve as a critical reference for the refinement of global RA intervention and treatment strategies.

## Methods

### Data Sources and Disease Definition

The Institute for Health Metrics and Evaluation (IHME), in collaboration with global partners, provides comprehensive and reliable data on global health trends. As part of this effort, the GBD study collected data on the burden of 371 diseases and injuries across 21 GBD regions and 204 countries and territories, spanning the years 1990 to 2021 (16). These publicly available data can be accessed freely from the following website: https://vizhub.healthdata.org/gbd-results/. Importantly, no further ethical approval is required for their use.

RA events were identified and classified using the International Statistical Classification of Diseases and Related Health Problems, 10th Revision (ICD-10). The specific ICD-10 codes used to define RA include M05.3, M05.8, M05.9, M06.0, M06.8, M06.9, and M08.0.

### Socio-demographic index

The SDI is a composite metric introduced by the IHME to measure the level of socio-economic development across regions. SDI incorporates three key dimensions: total fertility rate (among women aged under 25), mean years of schooling (for individuals aged 15 and older), and per capita income. Its values range from 0 (lowest level of development) to 1 (highest level of development). SDI has been extensively applied in GBD studies to analyze the influence of socio-economic factors on health outcomes and disease burden, providing a robust foundation for policymaking and resource allocation. In the GBD study, the 204 countries and territories were categorized into five SDI-based regions: Low, Low-middle, Middle, High-middle, and High (16).

### Data analysis

We utilized global, regional, and national data on elderly individuals (≥60 years old) with RA, focusing on incidence, prevalence, and DALYs in terms of cases and rates. The primary metrics calculated were the EAPC and percentage change.

DALYs is a metric used to quantify disease burden. It combines years of life lost (YLLs) due to premature death and years lived with disability (YLDs) due to illness. The formula is: *DALYs*=*YLLs* +*YLDs*. Where YLLs represent the years of life lost due to premature mortality, calculated as the difference between the age of death and the standard life expectancy for a given population. YLDs account for years lived with disability, weighted by the severity of the condition. DALYs provide a comprehensive measure of both mortality and morbidity, facilitating the prioritization of health interventions.

The EAPC is a widely adopted statistical measure used to quantify the annual trends of changes in specific indicators, such as incidence or mortality rates, over a defined period. It is derived using a linear regression model where the natural logarithm of time-series data is regressed on time to estimate the rate of change, expressed as an annual percentage. The EAPC value reflects the direction and magnitude of a trend: a positive value indicates an upward trend, a negative value suggests a downward trend and a value close to zero implies minimal or no significant change. EAPC is particularly suitable for long-term trend analysis because it smooths out short-term fluctuations, providing a clearer depiction of overall trends. In epidemiology and public health, particularly within the GBD framework, the EAPC is extensively applied to assess dynamic trends in diseases, injuries, and risk factors (17, 18).

Percentage change is mainly applied to measure the variations in prevalence cases, incidence cases, and DALYs. Specifically, percentage change (%) = [(cases in 2021 - cases in 1990)/cases in 1990] × 100% (19).

We utilized the Bayesian age-period-cohort model for predicting trends of RA in the elderly up to 2040. The BAPC model leverages Bayesian inference to address collinearity issues inherent in traditional age-period-cohort analyses while providing robust estimates of temporal patterns (20, 21).

The data were filtered and calculated using R (4.4.0). Visualizations were generated using ggplot2 (3.5.1).

## Results

### Global level

In 2021, the global prevalence cases of RA among the elderly was 7,919,136, comprising 5,687,570 women and 2,231,566 men. The overall prevalence rate was 726.9 (95% UI: 634.1, 834.8) cases per 100,000 population. Specifically, the prevalence rate for women was 969.9 (95% UI: 849.9, 1108.5) cases per 100,000 population, while for men, it was 445.7 (95% UI: 383.9, 519.8) cases per 100,000 population. Notably, the prevalence rate among women was 2.2 times higher than that among men. In 2021, the prevalence rate of RA among the elderly was significantly higher than that of RA across all age groups globally, with 227.1 (95% UI: 202.4, 257.3) cases per 100,000 population.

**Figure 1:**
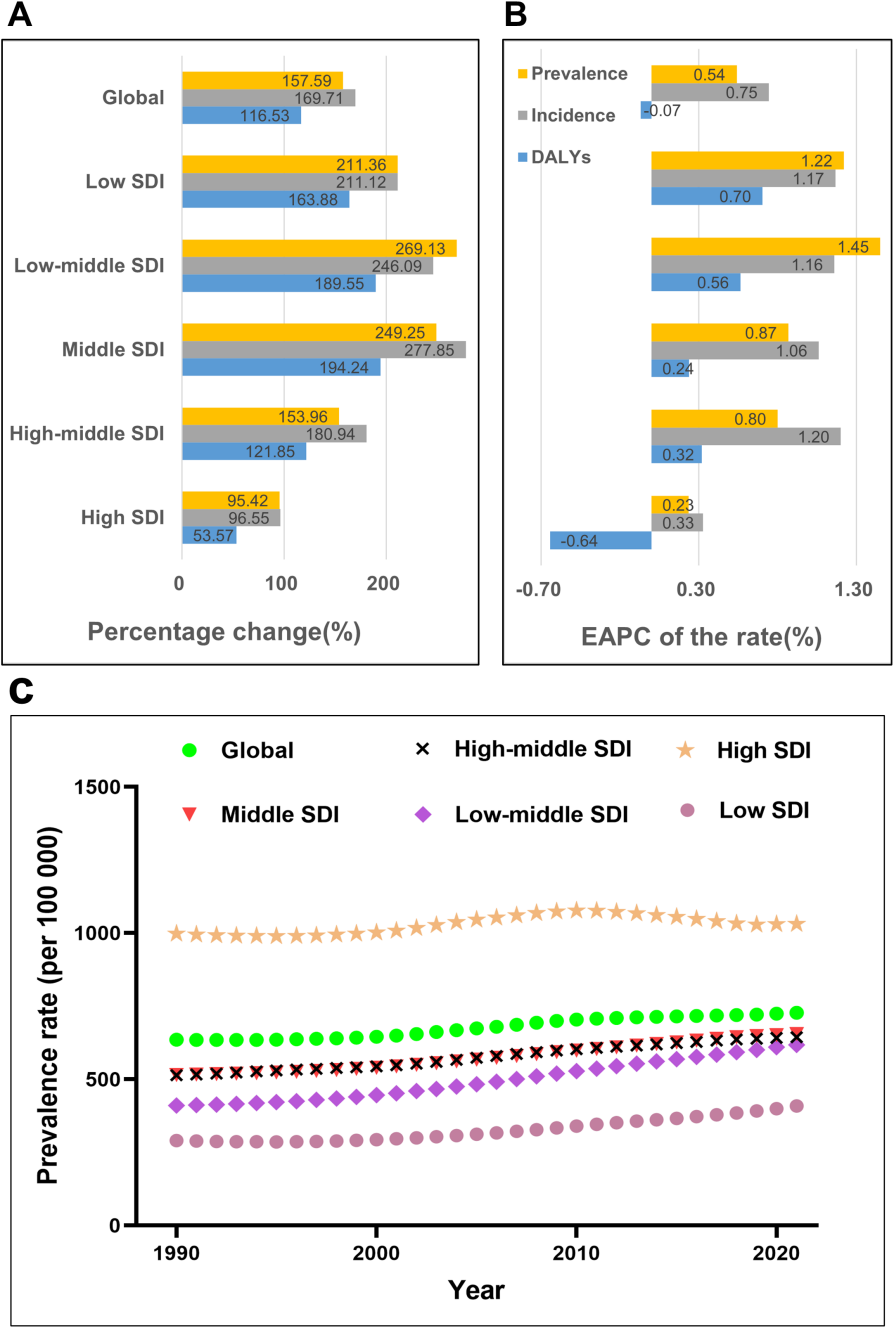
Temporal trends of the Rheumatoid Arthritis burden among the elderly globally and across the 5 SDI regions **(A)** Percentage change of cases of prevalence, incidence, and DALYs from 1990 to 2021. **(B)** The EAPC rates of prevalence, incidence, and DALYs from 1990 to 2021. **(C)** The prevalence rate from 1990 to 2021.

Between 1990 and 2021, the global prevalence cases, incidence cases, and DALYs of RA among the elderly increased significantly. However, the DALY rate was the only rate-based indicator that showed a slight decline. Specifically, from 1990 to 2021, the number of prevalence cases rose from 3,074,298 to 7,919,136, reflecting a percentage change of 157.59%. Similarly, incidence cases increased from 123,945 to 334,291, with a percentage change of 169.71%. DALYs grew from 715,789 to 1,549,877, representing a percentage change of 116.53%. Both the prevalence rate and incidence rate demonstrated upward trends, with EAPCs of 0.54 (95% CI: 0.5, 0.58) and 0.75 (95% CI: 0.7, 0.79), respectively. In contrast, the DALY rate exhibited a slight decline, with an EAPC of -0.07 (95% CI: -0.11, -0.03). These findings highlight that the global burden of RA among the elderly is intensifying. (**Figure 1**; **Supplementary Figures S1**–**S2**, **Table 1**; **Supplementary Tables S1–S2**).

**Table 1:** The prevalence of RA cases and rate among the elderly in 1990 and 2021, and the trends from 1990 to 2021.

Location	Prevalence cases		Percentage change(100%)	Prevalence rate		EAPC(95% CI)
	1990 (95% UI)	2021 (95% UI)		1990_per 100 000 (95% UI)	2021_per 100 000(95% UI)	
Global	3074297.8 (2663033.9, 3566184.7)	7919135.6 (6902538.9, 9101562.5)	157.59	635.5 (551.5, 735.5)	726.9 (634.1, 834.8)	0.54 (0.5, 0.58)
High SDI	1443686.2 (1264277.6, 1658853.2)	2821305.1 (2499105.3, 3199757.4)	95.42	997.5 (873.2, 1147)	1031.2 (911.7, 1171.9)	0.23 (0.16, 0.31)
High-middle SDI	652000.4 (565611, 751743.4)	1655819.3 (1448631.2, 1890716.4)	153.96	513.6 (446.2, 591.4)	644 (563.6, 735.1)	0.8 (0.77, 0.83)
Middle SDI	619211.7 (526768.2, 727526.6)	2162625.2 (1861774.4, 2520907.3)	249.25	514.8 (438.9, 603.5)	654.4 (564, 761.7)	0.87 (0.83, 0.9)
Low-middle SDI	282767.7 (237866, 337586.7)	1043788.4 (890691.9, 1230019.9)	269.13	409.9 (345.8, 488)	617.4 (527.9, 725.8)	1.45 (1.4, 1.51)
Low SDI	74010.4 (62732.2, 87385.5)	230439.2 (197348.9, 270529.6)	211.36	290.2 (246.7, 341.9)	408.6 (350.7, 478.4)	1.22 (1.1, 1.34)
High-income Asia Pacific	306750.6 (258776.8, 364702.9)	581259.5 (503030.9, 673504.1)	89.49	1202.8 (1015.4, 1428.8)	1000.3 (862, 1163.7)	-0.4 (-0.51, -0.28)
High-income North America	436947.1 (386272.8, 495876.5)	970351.6 (863514.1, 1091818.8)	122.08	936.7 (827.4, 1064)	1097 (975.7, 1234.8)	0.66 (0.6, 0.72)
Western Europe	730253.5 (638961.8, 838567.3)	1224007 (1077948, 1387092.3)	67.61	955.2 (834.8, 1098.5)	1029.9 (903, 1172.2)	0.32 (0.26, 0.38)
Australasia	43339.2 (38003.9, 49189.1)	104425.2 (91973.6, 118258.2)	140.95	1388.2 (1216.9, 1576.2)	1478.6 (1297.4, 1680.2)	0.28 (0.14, 0.43)
Andean Latin America	14278.5 (12735.4, 15957.3)	76234.9 (68312.6, 84815.9)	433.91	604 (539.6, 674.2)	1058.6 (949.1, 1177.2)	1.89 (1.85, 1.93)
Tropical Latin America	34353.1 (29523.9, 39808.7)	103052.4 (89810.3, 118478.9)	199.98	308.3 (265.3, 357.1)	315.7 (275.3, 362.8)	0.23 (0.18, 0.29)
Central Latin America	85105.1 (73693.2, 98363.8)	324017.9 (286604.2, 367374.9)	280.73	887.5 (768.9, 1025)	1047.3 (926.7, 1186.8)	0.53 (0.48, 0.58)
Southern Latin America	28269.2 (24840.7, 32259.7)	90017.3 (80582.6, 100579.9)	218.43	479.8 (422, 546.9)	802.4 (718.1, 897.1)	1.61 (1.54, 1.68)
Caribbean	10580.1 (9299.9, 12039.6)	31258.5 (27723.9, 35208.6)	195.45	333.3 (293.3, 378.9)	465.3 (412.7, 524.1)	1.08 (1.01, 1.15)
Central Europe	95700.3 (84567.3, 108337.5)	173188.8 (153779.3, 194534.4)	80.97	477.7 (422.3, 540.4)	581.6 (516, 653.7)	0.74 (0.7, 0.79)
Eastern Europe	123479.2 (109173.6, 139652.2)	191303.1 (170923.3, 213927.9)	54.93	326.6 (288.9, 369.1)	391.3 (349.7, 437.3)	0.63 (0.59, 0.68)
Central Asia	16793.1 (14888.3, 18964)	41125 (36892, 45830.3)	144.89	292 (259.2, 329.3)	403.4 (362.5, 448.7)	1.21 (1.01, 1.4)
North Africa and Middle East	32828.3 (28646.1, 37793.2)	146774.7 (130003.2, 165778.6)	347.10	161.4 (141, 185.4)	271.5 (240.7, 306.4)	1.81 (1.76, 1.86)
South Asia	340275.1 (281720.1, 412985.1)	1447349.9 (1215780, 1729249.4)	325.35	541.6 (450.1, 654.7)	827.9 (697.3, 986.3)	1.49 (1.42, 1.57)
Southeast Asia	46551.7 (39265.9, 55150.7)	183758.5 (157246.4, 214391.1)	294.74	158 (133.8, 186.4)	229.8 (197.4, 266.9)	1.2 (1.18, 1.23)
East Asia	666303.3 (564153.6, 789309.4)	2077439.9 (1781244.4, 2427412.7)	211.79	643.1 (545.6, 759.9)	745.5 (639.7, 870.3)	0.55 (0.52, 0.58)
Oceania	317.6 (265.5, 380.2)	925.9 (789.3, 1090.6)	191.53	92.1 (77.2, 109.9)	110.1 (94.2, 129.2)	0.5 (0.43, 0.56)
Western Sub-Saharan Africa	14084.4 (11701, 16827.2)	36960.5 (31220.5, 43605.9)	162.42	134.1 (111.8, 159.8)	166 (140.9, 195.2)	0.75 (0.61, 0.88)
Eastern Sub-Saharan Africa	21223.7 (18190.1, 24887.8)	53393.9 (46220.3, 61622.5)	151.58	250.1 (215, 292.3)	287.9 (249.7, 331.8)	0.49 (0.43, 0.54)
Central Sub-Saharan Africa	6866.9 (6006.7, 7840)	19947.4 (17495.4, 22789.7)	190.49	267 (234.2, 304)	333 (292.6, 379.5)	0.74 (0.62, 0.86)
Southern Sub-Saharan Africa	19997.8 (17506.8, 22893.2)	42343.7 (37146.4, 48561.1)	111.74	624.8 (546.8, 715.6)	612 (536.8, 701.6)	0.07 (-0.07, 0.2)

### SDI regional level

Overall, between 1990 and 2021, the prevalence cases, incidence cases, and DALYs of RA among the elderly increased significantly across the five SDI regions. However, among rate-based indicators, only the DALY rate in the High SDI region showed a decline. Notably, prevalence rates increased progressively with higher SDI levels.

In 2021, the High SDI region reported the largest number of elderly RA prevalence cases(2,821,305), accounting for approximately 36% of the global total, followed by the Middle SDI and High-middle SDI regions. Collectively, these three regions contributed 84% of the global prevalence cases. Interestingly, although the Low SDI and Low-middle SDI regions had fewer prevalence cases, they exhibited the fastest growth in prevalence rates during 1990–2021, with estimated annual percentage changes (EAPCs) of 1.45 (95% CI: 1.4, 1.51) and 1.22 (95% CI: 1.1, 1.34), respectively. Similar to prevalence cases, the High SDI region recorded the highest number of incident cases (114,994) in 2021, accounting for 34% of the global total. Combined, the Middle SDI, High-middle SDI, and High SDI regions represented 79% of global incident cases. The incidence rate was highest in the High SDI region and lowest in the Low SDI region. Between 1990 and 2021, the incidence rates in the Low SDI and Low-middle SDI regions increased the most rapidly, with EAPCs of 1.17 (95% CI: 1.09, 1.24) and 1.16 (95% CI: 1.13, 1.19), respectively. The trends in DALY cases and rates followed a similar pattern.

In summary, the High SDI region had the highest number of cases and rates, but its growth was the slowest, with a decline even observed in DALY rates. In contrast, the Low SDI and Low-middle SDI regions had lower prevalence and incidence cases and rates, but their prevalence and incidence cases and rates grew rapidly. (**Figure 1**; **Supplementary Figures S1**–**S2**, **Table 1**; **Supplementary Tables S1–S2**).

### GBD regional level

From 1990 to 2021, the prevalence cases and rates, incidence cases and rates, and DALYs increased across nearly all GBD regions. However, a few regions, including High-income Asia Pacific, Western Europe, Australasia, Central Europe, and Southern Sub-Saharan Africa, exhibited a decline in DALY rates, most of which are categorized as High SDI or High-middle SDI regions.

In 2021, the regions with the highest number of prevalence cases were East Asia, South Asia, Western Europe, High-income North America, and High-income Asia Pacific, most of which belonged to the Middle SDI to High SDI regions. In contrast, the regions with the highest prevalence rates were Australasia, High-income North America, Andean Latin America, Central Latin America, and Western Europe, with rates exceeding 1,000 cases per 100,000 population.

**Figure 2:**
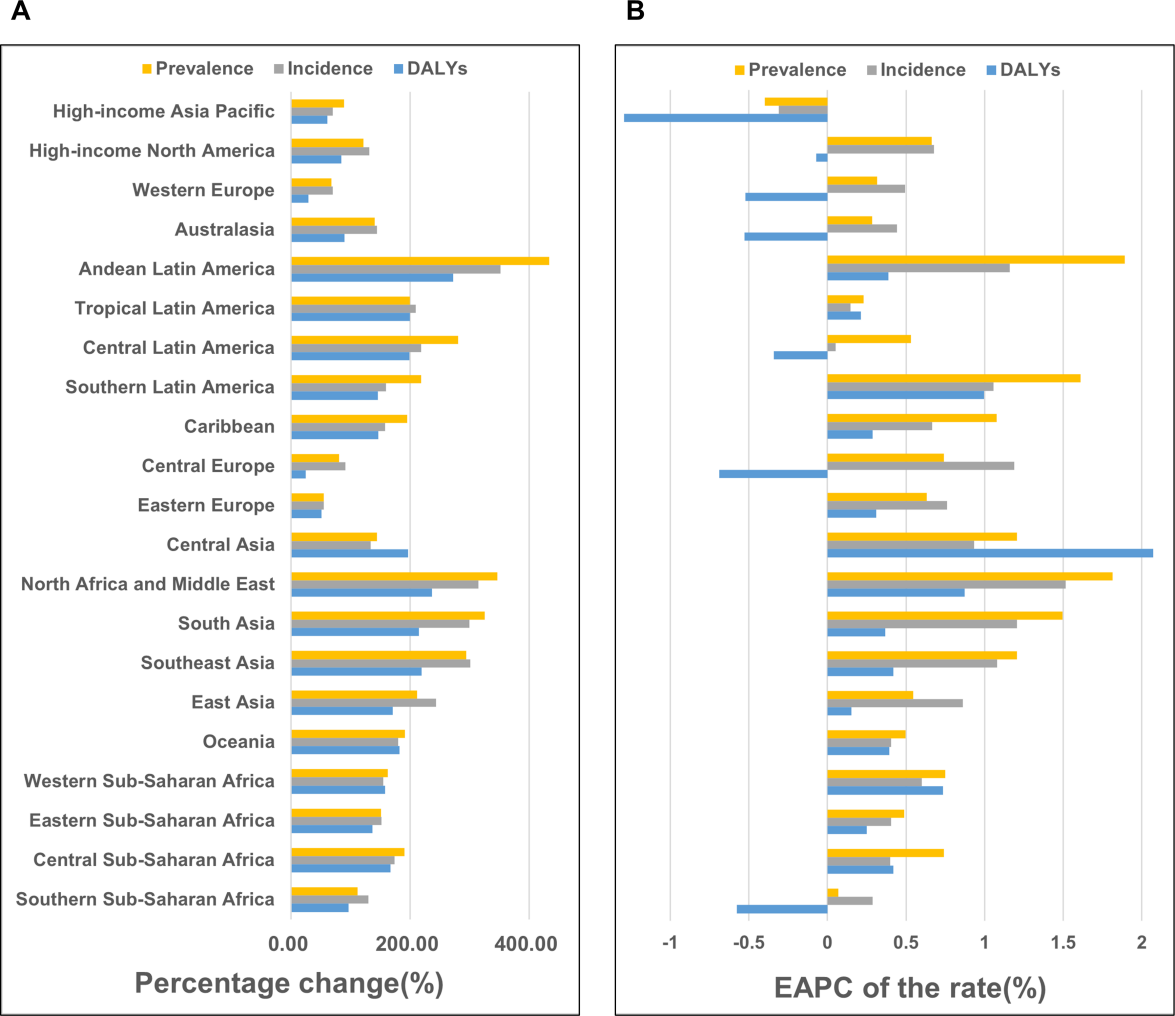
Temporal trends of the Rheumatoid Arthritis burden among the elderly across GBD regions **(A)** Percentage change of cases of prevalence, incidence, and DALYs from 1990 to 2021. **(B)** The EAPC rates of prevalence, incidence, and DALYs from 1990 to 2021.

The regions with the fastest growth in prevalence rates were Andean Latin America, North Africa and the Middle East, Southern Latin America, South Asia, and Southeast Asia. Notably, Andean Latin America, Southern Latin America, North Africa and the Middle East, South Asia, and Southeast Asia demonstrated significant increases in both prevalence cases and rates, reflecting a rising disease burden in these areas. (**Figure 2**; **Table 1**, **Supplementary Tables S1–S2**).

### Countries level

Between 1990 and 2021, the prevalence cases, incidence cases, and DALYs increased across all countries. Notably, Qatar, United Arab Emirates, Bahrain, Jordan, and Kuwait ranked among the top globally in percentage changes for all three indicators, reflecting a rapid rise in prevalence and incidence cases. However, the absolute number of prevalence cases in these countries remained relatively low in 2021, ranging from 246 to 1073 cases. These five countries, belonging to the High SDI region, showed a trend that contrasts with the overall pattern observed in other High SDI countries.

**Figure 3:**
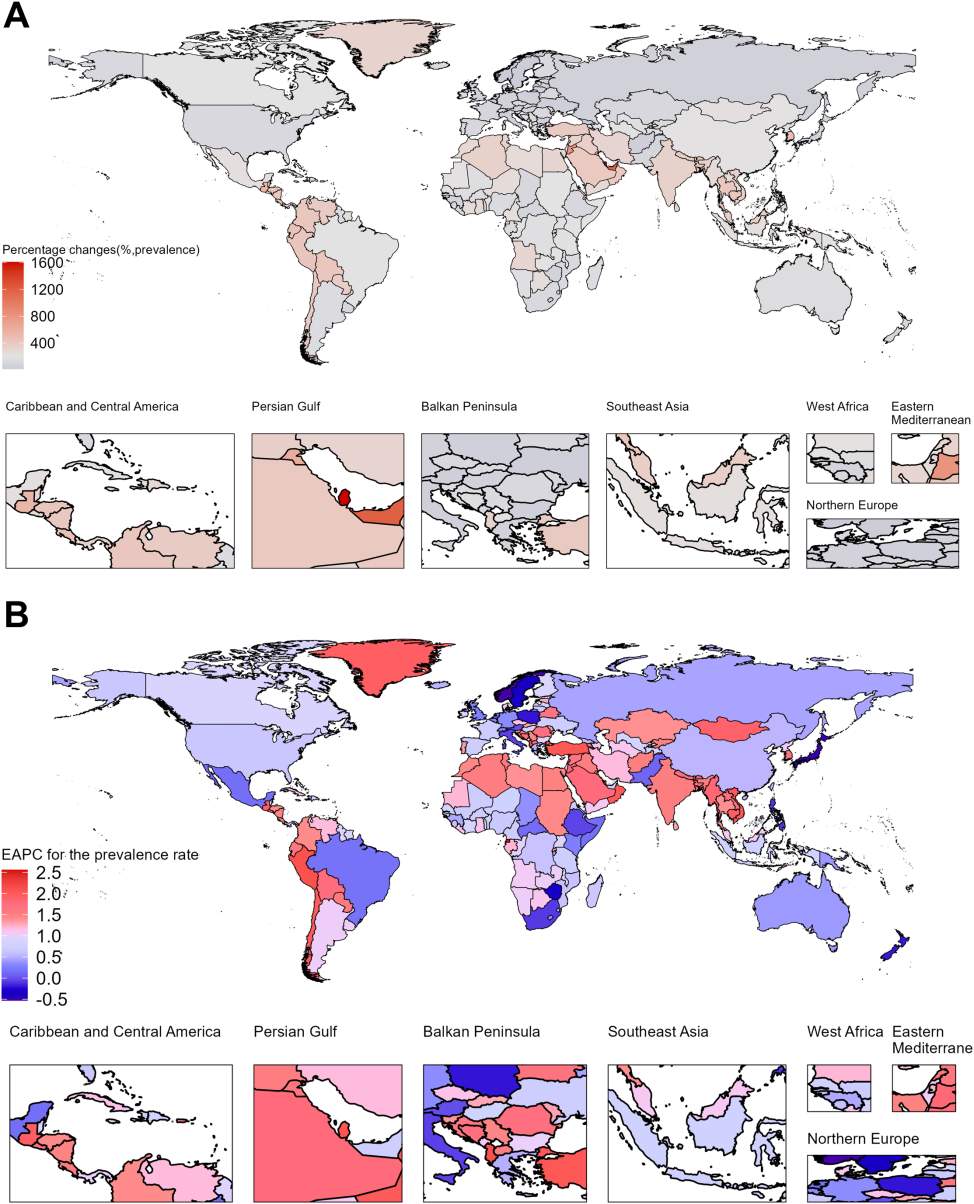
Temporal trends of Rheumatoid Arthritis burden in elderly across 204 countries. **(A)** Percentage change in prevalence cases from 1990 to 2021. **(B)** EAPC for prevalence rates from 1990 to 2021.

In 2021, the countries with the highest number of prevalence cases were China (2,012,064 cases), India (1,312,132 cases), Japan (473,300 cases), and the United Kingdom (275,624 cases). The countries with the highest prevalence rates were Ireland, Finland, the United Kingdom, and New Zealand, all exceeding 1628 cases per 100,000 population, and all of these countries belong to the High SDI region. In contrast, the countries with the fastest-growing prevalence rates were Equatorial Guinea, Cambodia, Guatemala, Mongolia, Myanmar, Bangladesh, and Nepal, with EAPCs ranging from 1.74% to 2.56%. These countries are categorized under the Low or Low-middle SDI regions. Additionally, a slight decline in prevalence rates was observed in only 11 countries, including Norway, Japan, Sweden, Poland, New Zealand, Austria, and Italy, with EAPCs ranging from -0.01% to -0.53%. All these countries belong to the High SDI region. A similar pattern was observed for incidence rates. Cambodia, Myanmar, Bangladesh, and Nepal exhibited rapid increases in incidence rates, with EAPCs exceeding 1.5%. Conversely, incidence rates slightly declined in only five countries: Japan, Sweden, Mexico, Zimbabwe, and Norway, with EAPCs ranging from -0.18% to -0.39%. Encouragingly, DALY rates showed a declining trend in 47 countries, most of which are located in the High or High-middle SDI regions. The three countries with the fastest declines in DALY rates were Poland, Norway, and Japan, with EAPCs of -1.85 (95% CI: -2.06, -1.63), -1.63 (95% CI: -1.73, -1.52), and -1.26 (95% CI: -1.36, -1.15), respectively. (**Figure 3**; **Supplementary Figures S3**–**S4**, **Supplementary Tables S3–S5**).

### Sex and age

**Figure 4:**
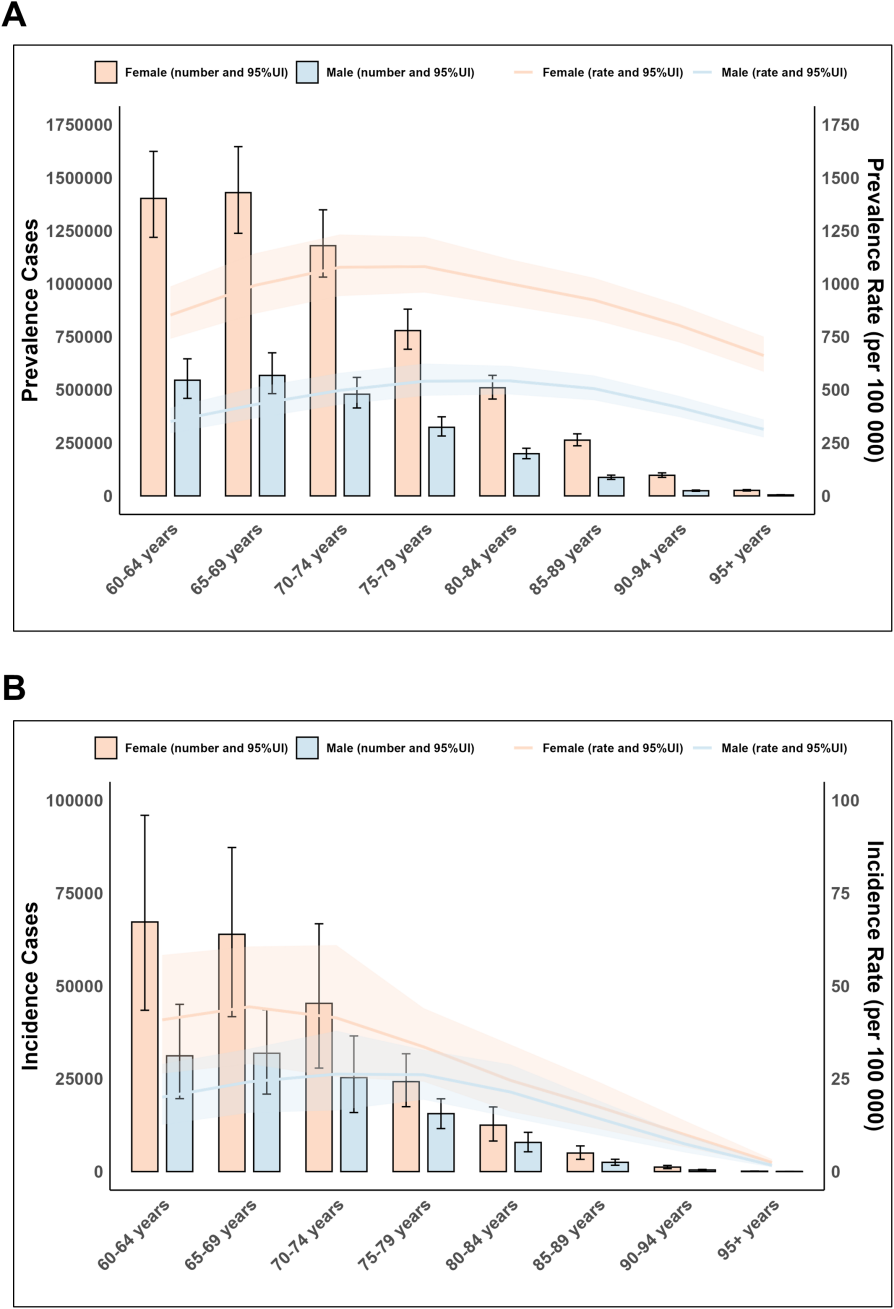
Age and sex trends of elderly Rheumatoid Arthritis globally in 2021. **(A)** Prevalence cases and rate. **(B)** Incidence cases and rate.

In 2021, the global prevalence cases, prevalence rates, and incidence rates of elderly RA exhibited a pattern of initially rising with increasing age and then declining, while the number of incidence cases consistently decreased. The prevalence cases and incidence rate were highest in the 65–69 age group, reaching 1,997,898.7 (95% UI: 1,722,725.3–2,323,991.5) and 34.7 cases per 100,000 population, respectively. The prevalence rate peaked in the 75–79 age group, reaching 711.3 (95% UI: 626.3–809.8) cases per 100,000 population. DALYs reached a peak in the 65–69 age group at 364,306.7 (95% UI: 285,628.8–466,155.2), followed by a rapid decline with advancing age. Overall, females exhibited higher prevalence and incidence cases/rates than males. The incidence rate ratio peaked at 2:1 in the 60–64 age group, then gradually decreased and converged, indicating a narrowing gender disparity in new RA cases among the elderly. (**Figure 4**; **Supplementary Figure S5**, **Supplementary Tables S6–S8**).

### Decomposition analysis

**Figure 5:**
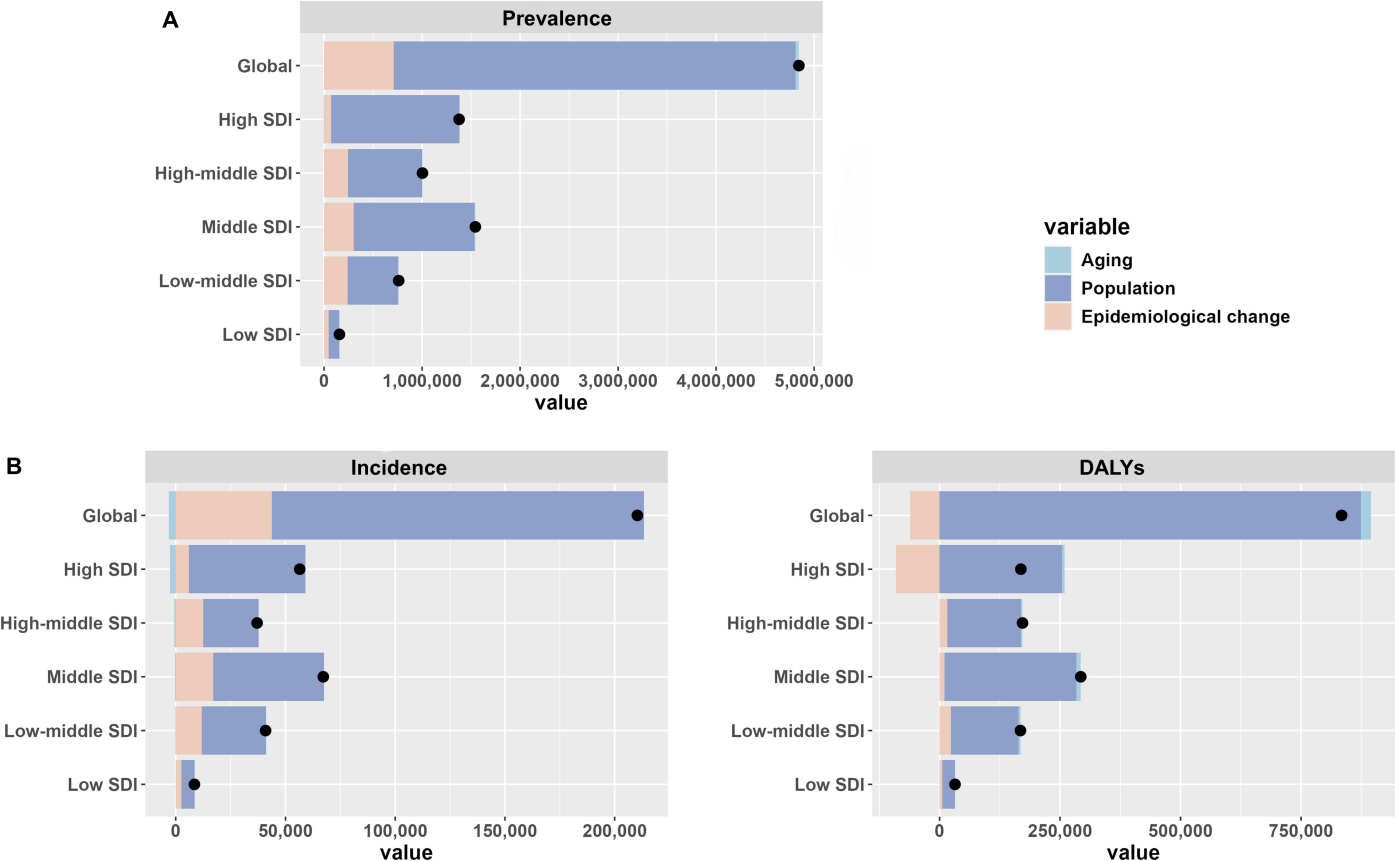
Decomposition analysis of the global burden of elderly RA in 2021. **(A)** Prevalence **(B)** Incidence **(C)** DALYs.

Decomposition analysis indicated that the disease burden increased globally and across the five SDI regions, primarily driven by global population growth. For example, 84.66% of the global prevalence case burden was attributable to population growth, 0.71% to population aging, and 14.63% to changes in RA epidemiology. Notably, as previously mentioned, DALY rates declined in 47 countries, nearly all of which are in the High SDI region. This decline was largely due to changes in RA epidemiology, which reduced the DALY burden in the High SDI region by -53.58%. However, population growth remained the dominant factor contributing to the increase in DALYs in the High SDI region, accounting for 150.88%. (**Figure 5**; **Supplementary Table S9**).

### Projections for the next 20 years

**Figure 6:**
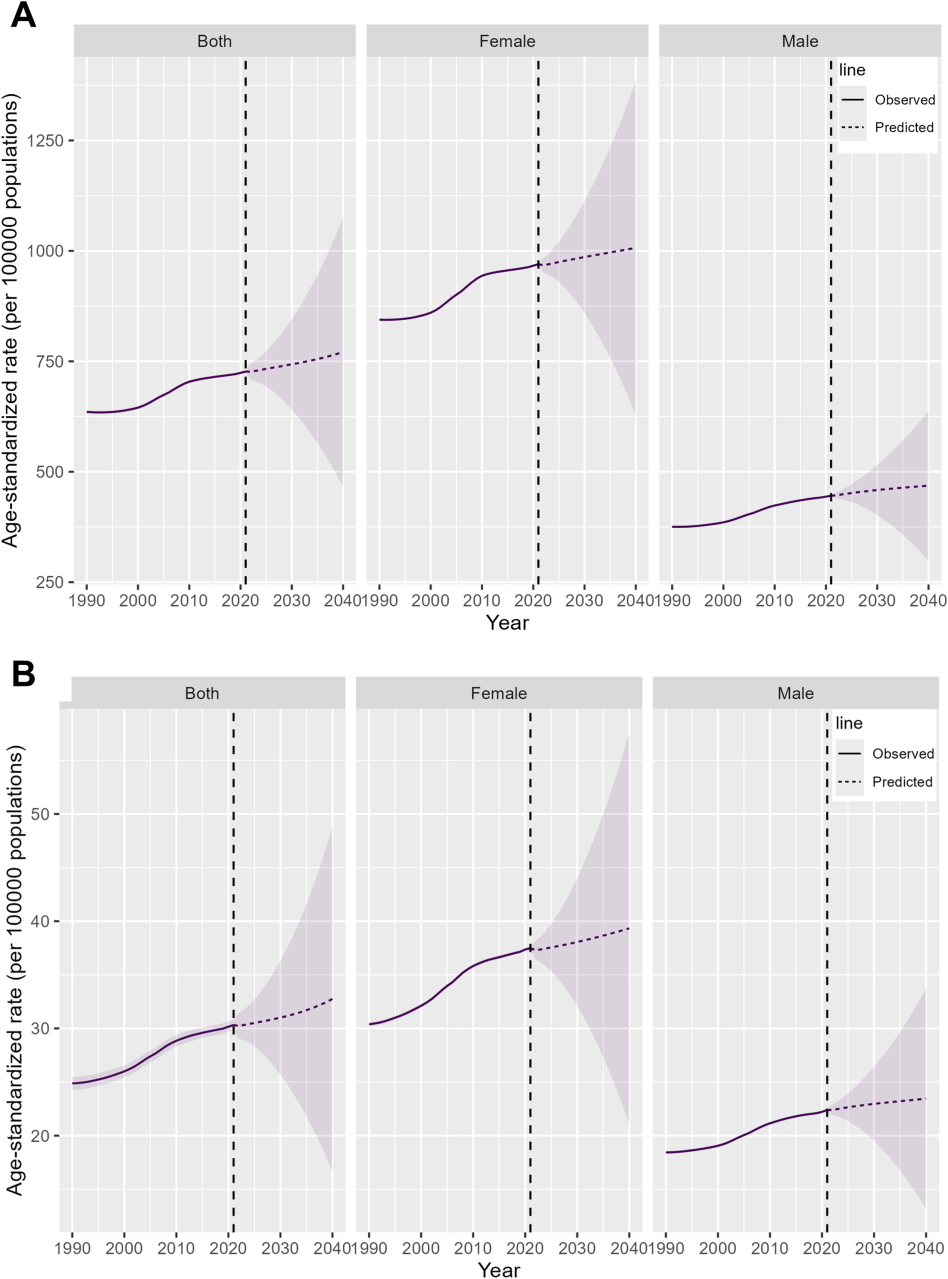
Projections of the global burden of elderly RA for 2021 to 2040. **(A)** Prevalence rate **(B)** Incidence rate.

In the next 20 years, the prevalence cases, prevalence rate, incidence cases, incidence rate, and DALYs of elderly RA are projected to rise, while the DALY rate is expected to decline. Specifically, between 2021 and 2040, the number of prevalence cases is projected to increase from 7,919,136 to 13,916,557, with the prevalence rate rising from 727 to 771 cases per 100,000 population. During the same period, incidence cases are expected to grow from 334,290 to 577,258, and the incidence rate will increase from 30 to 33 cases per 100,000 population. DALYs are projected to rise from 1,549,877 to 2,496,929, whereas the DALY rate will decrease from 143 to 137 per 100,000 population, with the rate of decline among males being comparatively slower. (**Figure 6**; **Supplementary Figure S6**, **Supplementary Table S10-S12**).

## Discussion

Aging is associated with chronic “sterile” systemic inflammation, a phenomenon known as “inflammaging” (22). Although this inflammation is typically at low levels, it contributes to the development and progression of RA. Additionally, with aging, the adaptive immune system declines, and the functions of T cells and B cells decrease, leading to weakened immune responses and increased susceptibility to diseases (23). As a result, elderly RA patients are more prone to systemic complications (24, 25). Furthermore, population aging and increased life expectancy have led to a continuous rise in the prevalence of elderly RA, further exacerbating the disease burden. Therefore, addressing this issue is crucial for public health policy development. This study is the first global report on the disease burden of elderly RA.

At the global level, from 1990 to 2021, the prevalence cases, incidence cases, and DALYs of elderly RA increased significantly, while the DALY rate showed a slight decline, indicating an overall worsening disease burden. Notably, in 2021, females accounted for a higher number of prevalence cases, with a prevalence rate approximately 2.2 times that of males, consistent with previous studies reporting a higher prevalence of RA in females. A 2020 disease burden analysis reported that the global prevalence rate of RA across all age groups was 208.8 cases per 100,000 population, which is substantially lower than the prevalence rate observed in the elderly population (726.9 cases per 100,000 population). Additionally, the percentage change in prevalence cases for all age groups (121%) was significantly lower than that for elderly patients (157.59%) (26). These findings indicate that, compared to the total population, elderly patients experience a higher prevalence rate, faster growth in prevalence cases, and a greater disease burden.

Analyzing data from the SDI and GBD regional levels, the High SDI region and its corresponding GBD regions show the highest prevalence and incidence cases and rates, which are potentially linked to multiple lifestyle and dietary factors. These include obesity, coffee consumption, high intakes of salt, fat, and sugar, excessive red meat consumption, and high-protein diets (27–29). In contrast, the Low SDI region currently has relatively low prevalence and incidence cases and rates; however, these metrics are increasing rapidly. This trend may be attributed to smoking, occupational exposure to silica, and air pollution (30, 31). Smoking triggers the activation of local respiratory epithelial cells, leading to the production of pro-inflammatory factors and enhanced recruitment of immune cells. At the same time, inhaled silica is engulfed by macrophages, promoting the release of TNF-α and IL-1β, thereby amplifying the inflammatory response (32).

Air pollution is a major environmental issue in South Asia, Southeast Asia, the Middle East, and Mongolia, with key sources including coal combustion, vehicle exhaust, and industrial emissions. In the Middle East, desert dust is also a contributing factor (33–35). At the national level, we observed rapid increases in prevalence and incidence cases in Middle Eastern countries (Qatar, United Arab Emirates, Bahrain, Jordan, Kuwait), and rapid growth in prevalence and incidence rates in South/Southeast Asian countries (Cambodia, Myanmar, Bangladesh, Nepal) and Mongolia. We consider this to be related to air pollution, as air pollution is one of the risk factors for RA (36). Fortunately, we observed declines in prevalence rates in 11 countries and DALY rates in 47 countries, most of which belong to the High SDI region, including Norway, Sweden, and Japan. This may be attributed to early disease detection, timely and sustained treatment, and the widespread use of disease-modifying antirheumatic drugs (DMARDs) in high-income regions (26, 37). For example, a 2015 study in Norway demonstrated that the use of DMARDs can improve long-term outcomes for RA patients and reduce joint damage (38). This suggests that in countries with high incidence and prevalence rates, early diagnosis and treatment should be encouraged to prevent joint damage progression in 90% of early RA patients, thereby reducing the disease burden (39).

We found that the incidence rate of elderly female patients remained higher than that of males, with a ratio of approximately 2:1 at ages 60–64. However, this gender difference in incidence rates gradually diminished with advancing age. Previous studies have reported that RA is more prevalent in females, with a ratio of 2–4:1 (40), potentially linked to the influence of sex hormones. Specifically, androgens negatively regulate the development, proliferation, and maturation of B lymphocytes, suppressing antibody production and secretion. They also reduce monocyte secretion of inflammatory cytokines, including Tumor Necrosis Factor-alpha (TNF-α), Interleukin-1 (IL-1), and Interleukin-1 (IL-6), thereby mitigating immune-inflammatory responses (41–43). Additionally, Yang et al., in a large cohort study involving 105,303 prostate cancer patients, found that the use of androgen inhibitors increased the risk of RA by an average of 23%. When used for more than 13 months, the risk rose to 33% (44). The role of estrogen in RA is more complex, as it exerts both stimulatory and inhibitory effects on the immune system. An imbalance between estrogen’s pro-inflammatory effects and androgen’s anti-inflammatory effects is associated with an increased risk of RA (45). Notably, women around age 60 enter menopause, during which estrogen levels decrease significantly, yet the incidence rate peaks. Our findings are consistent with this observation, as the incidence rate in females aged 60–64 is the highest. This phenomenon may be explained by the low post-menopausal estrogen levels enhancing the production of pro-inflammatory cytokines (46, 47). Furthermore, local synovial hormones, such as progesterone, aldosterone, and growth hormone, contribute to the development of RA (48). Finally, it is important to note that, regardless of gender, the incidence rate gradually decreases with age, which may be attributed to the declining functionality of the immune system (49).

Decomposition analysis revealed that the rapid increase in the global disease burden of elderly RA is primarily driven by the growth of the elderly population. A 2021 global population analysis showed that the total population had reached 7.89 billion, a significant increase from 2.52 billion in 1950. Regions experiencing the fastest population growth were primarily Sub-Saharan Africa, South Asia, Southeast Asia, East Asia, and Oceania, with particularly pronounced increases in the elderly population. Between 2000 and 2021, 188 countries and regions worldwide experienced growth in the proportion of individuals aged 65 and above. This trend was observed in all high-income countries, as well as in regions such as South Asia, Southeast Asia, East Asia, Latin America, and the Caribbean (50). As SDI increases, life expectancy rises in 2021 (50), with values of 64.9, 69.9, 73.1, 75.7, and 79.9 years in regions with Low, Low-middle, Middle, High-middle, and High SDI, respectively. This partly explains why the high SDI region bears the greatest burden. These findings underscore the critical relationship between addressing population growth and mitigating the disease burden.

Our study has several limitations. First, despite our efforts to perform a comprehensive analysis, the GBD data were sourced from various regions, and potential inconsistencies in the data may have introduced bias. Additionally, for the 20-year projections, while rigorous statistical models were employed, external factors could influence the actual outcomes, potentially leading to some degree of bias.

## Conclusions

Over the past 32 years, the global burden of RA among the elderly has significantly increased. High SDI regions bear the heaviest burden, while Low and Lower-middle SDI regions are experiencing rapid increases in burden. In 2021, females had a higher disease burden than males, with the 65–69 age group being the most affected. The burden is expected to intensify over the next 20 years. Early diagnosis and treatment of RA in the elderly are crucial for reducing adverse outcomes and the overall burden.

## Data Availability

The original contributions presented in the study are included in the article/**Supplementary Material**. Further inquiries can be directed to the corresponding author.
