# Supplementary material for: Global, regional, and national burden and trends of rheumatoid arthritis among the elderly population: an analysis based on the 2021 Global Burden of Disease study
Source: Front Immunol. 2025 Apr 15;16:1547763. doi: 10.3389/fimmu.2025.1547763 (PMC12037513; doi:10.3389/fimmu.2025.1547763)
Supplement: Supplementary file 2 [file DataSheet2.docx]

Supplementary
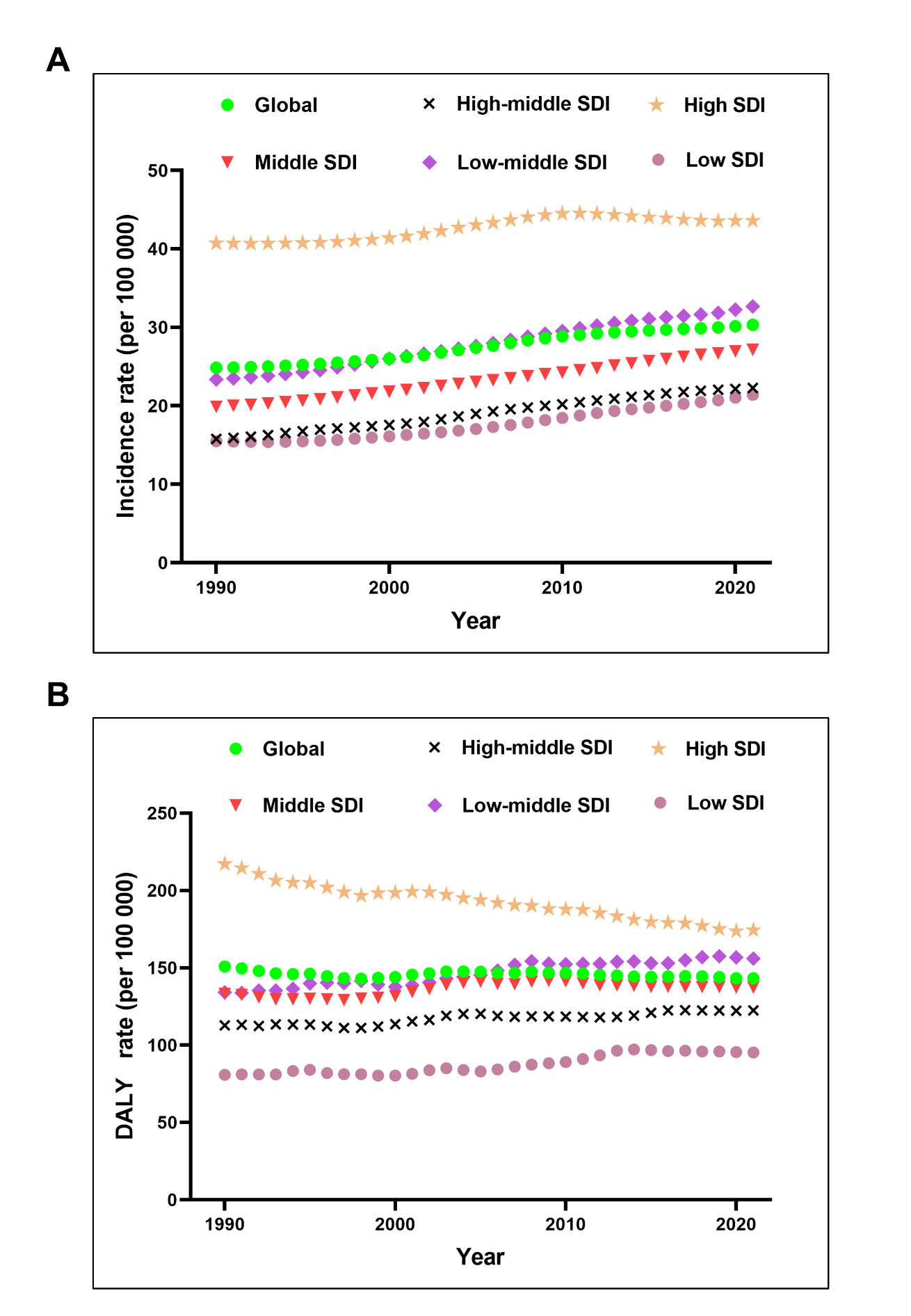
Figure S1. Temporal trends of the Rheumatoid Arthritis burden among the elderly globally and across the 5 SDI regions (A) The incidence rates from 1990 to 2021. (B) The DALY rate from 1990 to 2021.


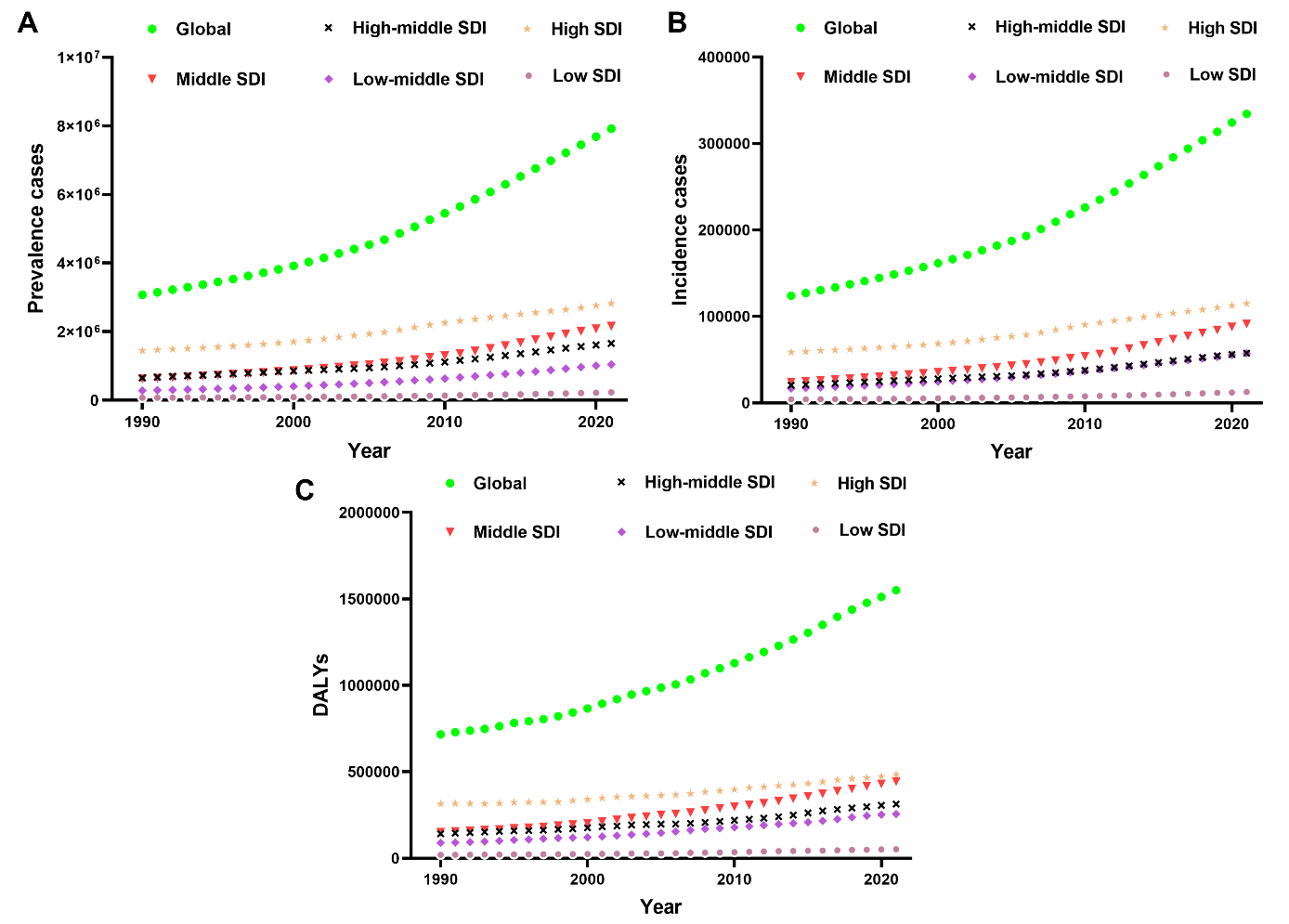


Supplementary Figure S2. Temporal trends of the Rheumatoid Arthritis burden among the elderly globally and across the 5 SDI regions (A) The prevalence cases from 1990 to 2021. (B) The incidence cases from 1990 to 2021. (C)The DALYs from 1990 to 2021.

Supplementary
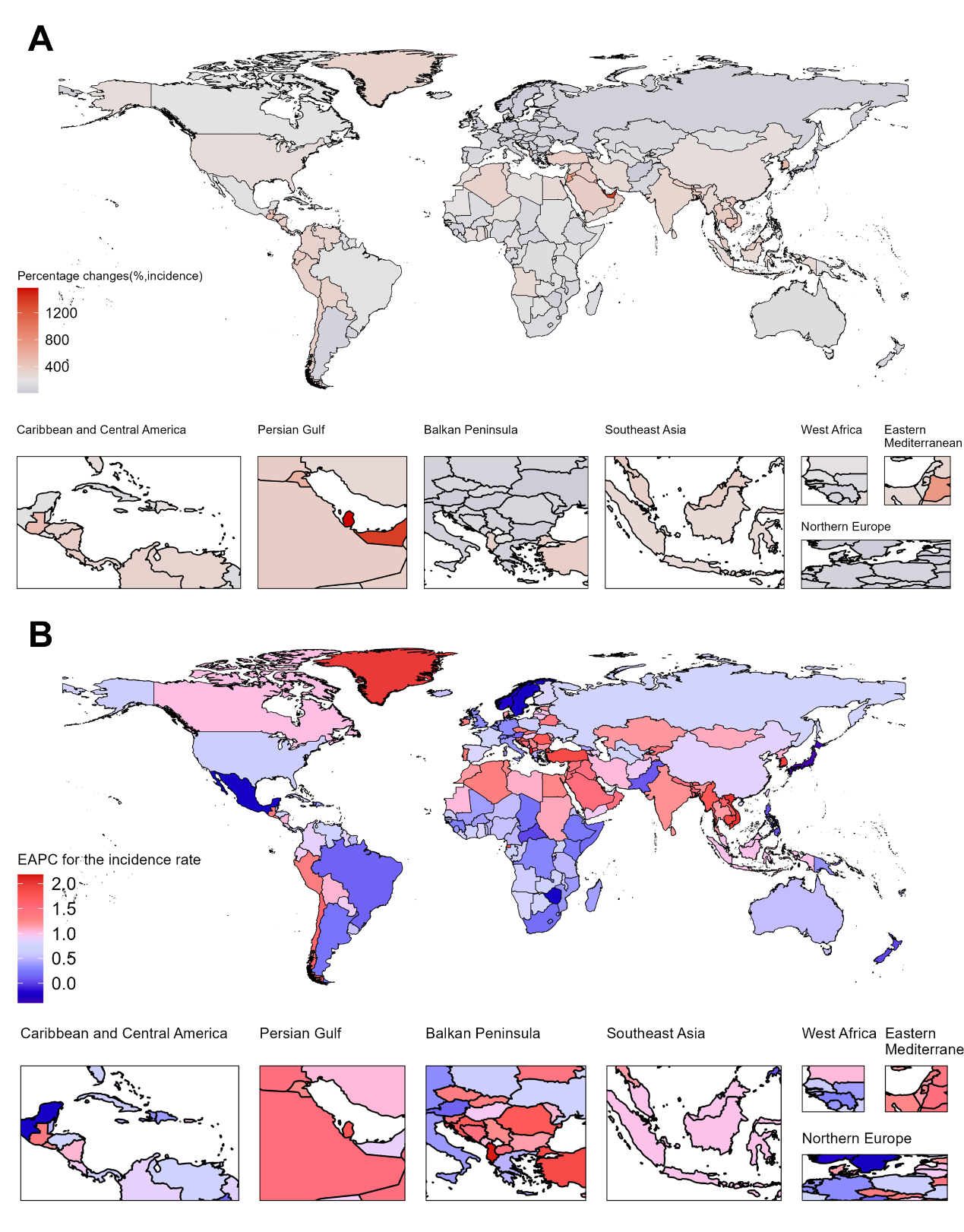
Figure S3. Temporal trends of Rheumatoid Arthritis burden in elderly across 204 countries. (A) Percentage change of incidence cases from 1990 to 2021. (A) EAPC for incidence rate from 1990 to 2021.

Supplementary
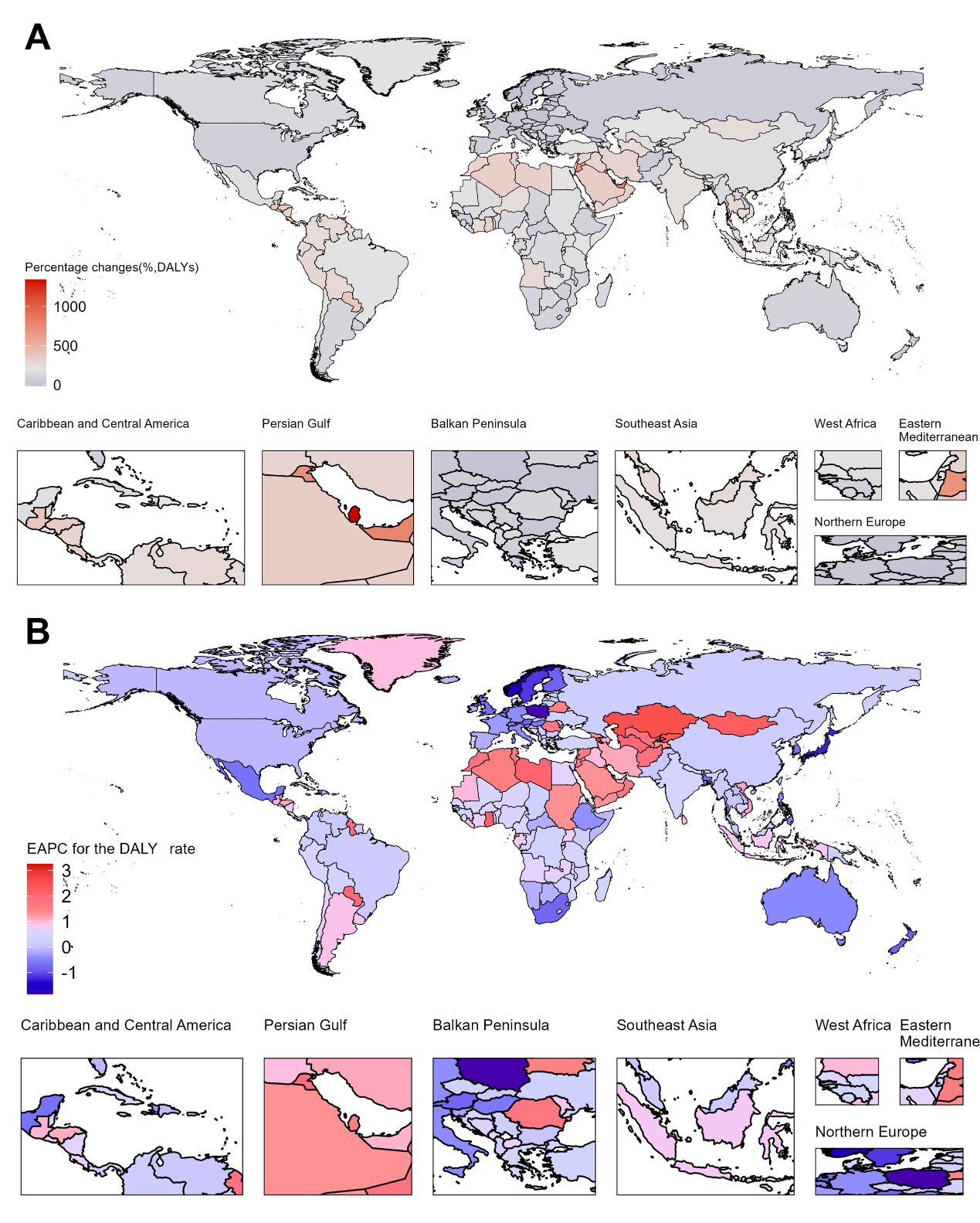
Figure S4. Temporal trends of Rheumatoid Arthritis burden in elderly across 204 countries. (A) Percentage change of DALYs from 1990 to 2021. (A) EAPC for DALY rate from 1990 to 2021.


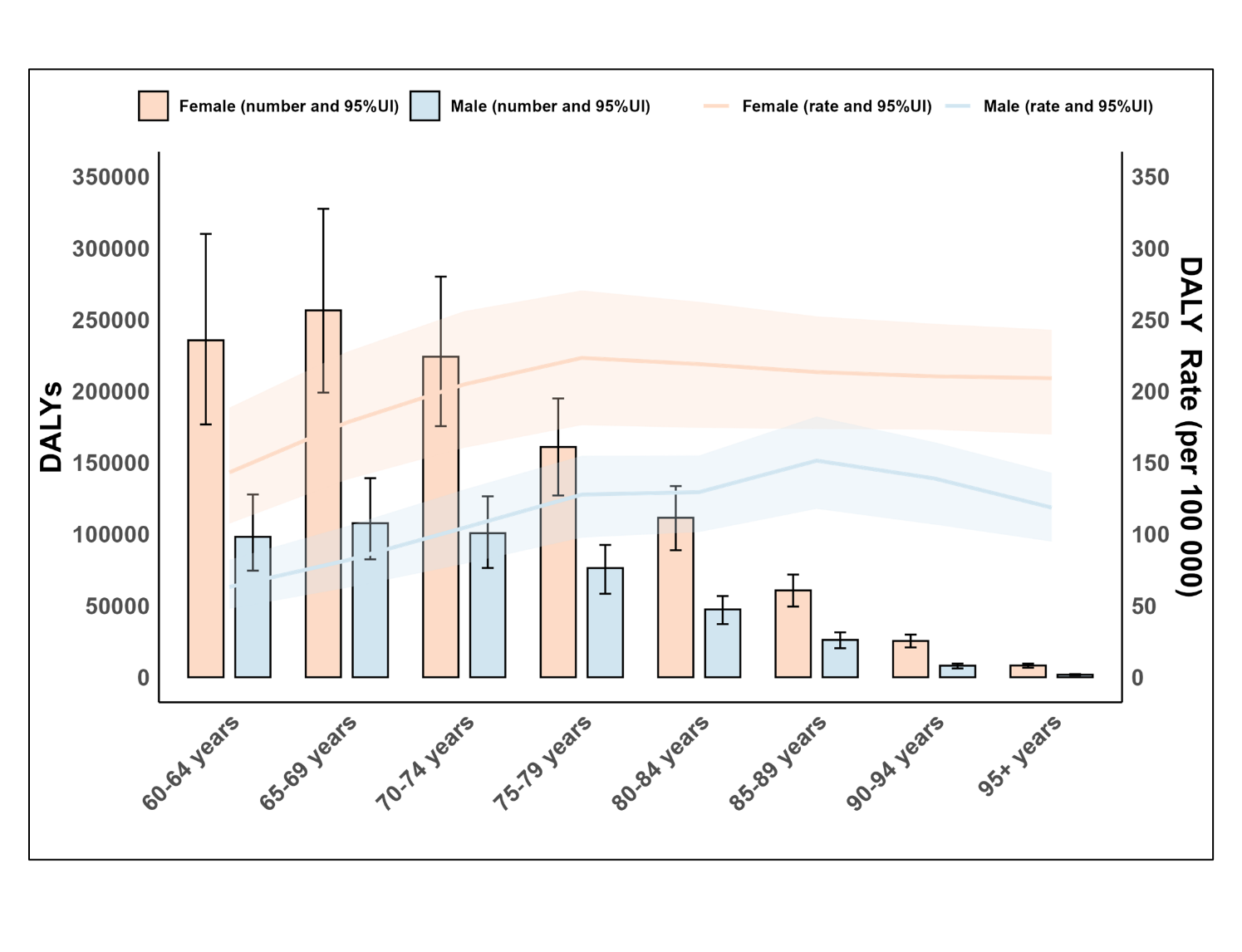


Supplementary Figure S5. Age and sex trends of elderly RA globally in 2021: DALYs and DALY rate.


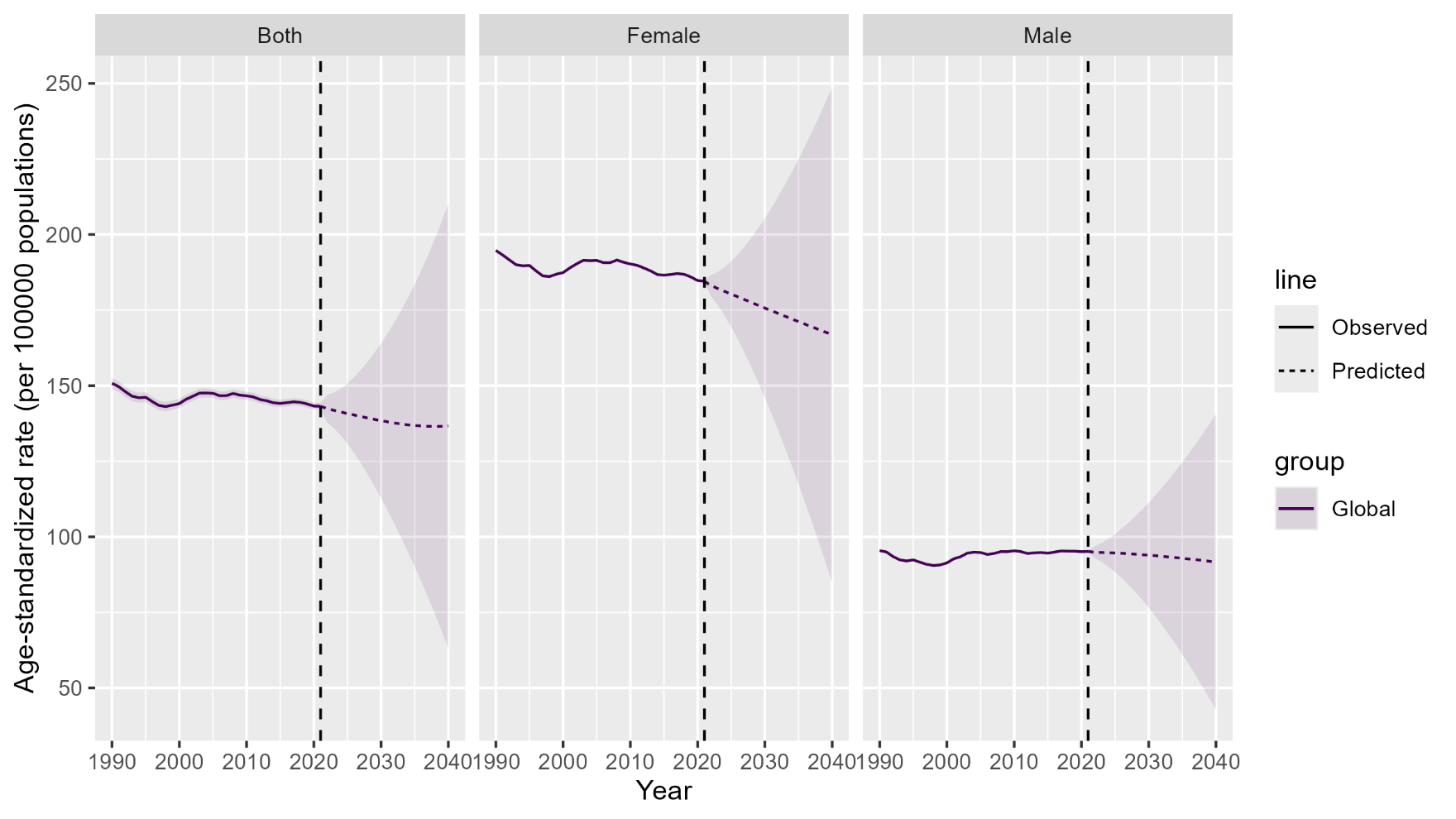


Supplementary Figure S6. Projections of the global burden of elderly RA for 2021 to 2040: DALY rate.
